# Non-Invasive Monitoring of Intracranial Pressure Pulse Waves from Closed Eyelids in Patients with Normal-Tension Glaucoma

**DOI:** 10.3390/medicina61040566

**Published:** 2025-03-22

**Authors:** Laimonas Bartusis, Solventa Krakauskaite, Ugne Kevalaite, Austeja Judickaite, Arminas Zizas, Akvile Stoskuviene, Edvinas Chaleckas, Mantas Deimantavicius, Yasin Hamarat, Fabien Scalzo, Kristina Berskiene, Ingrida Januleviciene, Arminas Ragauskas

**Affiliations:** 1 Health Telematics Science Institute, Kaunas University of Technology, LT-51423 Kaunas, Lithuania; solventa@mail.com (S.K.); edvinas.chaleckas@ktu.lt (E.C.); m.deimantavicius@gmail.com (M.D.); yasin.hamarat@ktu.lt (Y.H.); arminas.ragauskas@ktu.lt (A.R.); 2 Eye Clinic, Lithuanian University of Health Sciences, LT-50161 Kaunas, Lithuania; ugne.kevalaite@lsmu.lt (U.K.); austeja.judickaite@lsmu.lt (A.J.); arminas.zizas@lsmu.lt (A.Z.); akvile.stoskuviene@lsmu.lt (A.S.); ingrida.januleviciene@kaunoklinikos.lt (I.J.); 3 Department of Neurology, Medical Academy, Lithuanian University of Health Sciences, LT-44037 Kaunas, Lithuania; 4 Department of Neurology and Computer Science, University of California, Los Angeles (UCLA), Los Angeles, CA 90095, USA; fab@cs.ucla.edu; 5 Department of Sports Medicine, Lithuanian University of Health Sciences, LT-50161, Kaunas, Lithuania; kristina.berskiene@lsmu.lt

**Keywords:** normal-tension glaucoma, non-invasive monitoring, intracranial pressure pulse waves, glaucoma screening

## Abstract

*Background and Objectives*: Normal-tension glaucoma (NTG) is a subtype of primary open-angle glaucoma characterized by progressive optic nerve damage despite intraocular pressure (IOP) remaining within the normal range. The underlying pathophysiology of NTG remains incompletely understood, and its diagnosis is often delayed due to the lack of a definitive screening tool. This study aimed to evaluate differences in intracranial pressure pulse wave amplitude recorded from closed eyelids between NTG patients and control subjects using a novel non-invasive monitoring technology. *Materials and Methods*: A cross-sectional observational study was conducted, enrolling NTG patients and age-matched controls. Intracranial pressure pulse wave signals were recorded from closed eyelids using the ’Archimedes’ 02 device, which employs a highly sensitive digital pressure sensor and hydromechanical coupling for signal transmission. The amplitude of recorded intracranial pressure pulse waves was analyzed and compared between groups. Statistical analyses were performed using IBM SPSS Statistics 30.0, with significance set at *p* < 0.05. *Results*: A total of 140 participants were enrolled, including 68 NTG patients and 72 controls. After applying exclusion criteria, 63 NTG patients and 68 controls were included in the final analysis. The median intracranial pressure pulse wave amplitude was significantly higher in NTG patients (0.1326 a.u.) than in controls (0.0889 a.u.), with *p* = 0.01. *Conclusions*: These findings suggest that intracranial pressure pulse wave monitoring may serve as a potential biomarker for NTG. Further studies are needed to determine the diagnostic accuracy, sensitivity, and specificity of this technology for NTG detection.

## 1. Introduction

Glaucoma is a progressive optic neuropathy involving the degeneration of retinal ganglion cells and damage to the optic nerve head, leading to visual field deterioration and, if left untreated, irreversible blindness [1,2,3]. Glaucoma is categorized based on its anatomical and pathophysiological characteristics, with open-angle and angle-closure representing the two main subtypes [4]. Differentiating between open-angle and angle-closure glaucoma relies on a thorough evaluation of the anterior chamber angle using gonioscopy [4,5]. Open-angle glaucoma is characterized by a wide angle between the iris and the cornea; however, aqueous humour drainage is impaired, leading to a gradual increase in intraocular pressure (IOP) and the progression of the disease, often without noticeable symptoms [6,7,8]. In closed-angle glaucoma, the drainage angle between the iris and the cornea becomes closed, commonly due to the iris pushing forward. This usually leads to a rapid increase in IOP and the development of symptoms such as ocular pain, redness, decreased vision, and headaches [6,9]. Glaucoma is also classified as primary or secondary [4,10]. When the disease occurs without an identifiable cause, both open-angle and closed-angle glaucoma are termed primary glaucoma [4]. Secondary glaucoma describes any type of glaucoma caused by an identifiable factor leading to increased IOP and subsequent optic nerve damage [4].

Primary open-angle glaucoma (POAG) is the most common form of glaucoma, with an estimated 52.68 million cases among the adult population aged 40–80 years in 2020 [11]. It is projected that the global prevalence of glaucoma will reach 111.8 million cases by 2040, driven by factors such as population ageing and growth [12].

Normal-tension glaucoma (NTG) is a subtype of primary open-angle glaucoma characterized by glaucomatous optic nerve damage occurring in patients whose IOP consistently remains below 21 mmHg [4,13,14]. The pathogenesis of NTG is poorly understood and remains under investigation. Recent findings suggest that impaired ocular blood flow, an increased translaminar pressure gradient, disrupted cerebrospinal fluid circulation, neurodegenerative disorders, oxidative stress, genetic factors, and abnormal biomechanics of the lamina cribrosa contribute to the etiology of the condition [15,16,17,18]. The proportion of NTG among primary open-angle glaucoma cases varies widely, ranging from 30% in an Italian study to as high as 92% in a Japanese investigation, and is influenced by ethnicity [19,20,21]. However, the proportion of NTG among patients with POAG in glaucoma clinics worldwide is generally less than 30% [21]. These figures highlight global underdiagnosis, letting NTG progress go unchecked and potentially causing blindness [21].

There is a growing demand for innovative screening techniques to enable early glaucoma diagnosis. A group of researchers from Canada has developed a sophisticated Fourier-domain optical coherence tomography system to measure subtle pulsations in ocular structures [22]. In a study with glaucoma patients, they found that the amplitude of pulsatility in ocular elements, such as the axial distance between the retina and the optic disc cup, is significantly greater in glaucoma patients compared to controls [23].

We recently developed a novel, non-invasive method and system for monitoring intracranial pressure waves [24]. This technology captures pulsations as pressure signals through the closed eyelid using a highly sensitive pressure sensor and hydromechanical coupling. To ensure efficient signal transmission from the pulsating outer ocular structures to the digital pressure sensor, two chambers filled with a non-compressible liquid were designed—one for each eye. A thin elastic film acts as a sealing layer to prevent direct contact between the closed eyelid and the fluid. In this study, we aimed to evaluate differences in the amplitude of pressure pulse waves recorded with this technology between patients with normal-tension glaucoma and control subjects.

## 2. Materials and Methods

### 2.1. Study Design and Participants

We conducted a cross-sectional observational study in accordance with the STROBE recommendations [25]. The Kaunas Regional Biomedical Research Ethics Committee approved the study (Approval No. BE-2-15, dated 2024-02-10), and it was conducted in compliance with the ethical principles outlined in the Declaration of Helsinki [26]. The study was also registered on ClinicalTrials.gov (Registration No.: NCT06443411).

Normal-tension glaucoma patients and control group subjects were recruited from the Hospital of the Lithuanian University of Health Sciences Kaunas Clinics between 22 April 2024 and 3 February 2025.

Participants in the study group were patients diagnosed with NTG before our study, confirmed by an ophthalmologist based on characteristic glaucomatous changes in the optic nerve head, visual field defects, an open anterior chamber angle, and an intraocular pressure of IOP ≤ 21 mmHg on the daily curve at the time of diagnosis, without the use of antiglaucoma medications. At the time of our study, some NTG patients were not receiving antiglaucoma medication, while others had initiated treatment following the confirmation of their diagnosis by an ophthalmologist. Additionally, the IOP on the day of the study examination was ≤21 mmHg in all participants, regardless of their antiglaucoma treatment status.

The control group consisted of subjects without glaucoma (i.e., those with normal-appearing optic nerve heads, no retinal nerve fibre layer [RNFL] thinning, and normal visual fields). Participants in the control group also had no acute or chronic uncompensated conditions that could influence study outcomes. Matching between the NTG and control groups was performed based on age and anthropometric parameters.

Exclusion criteria for both groups were as follows:

1. Refusal to participate;
2. Age under 25 or over 85 years;
3. Pregnancy or breastfeeding;
4. Allergy or sensitivity to local anesthetics;
5. Eye diseases that could distort study results;
6. History of orbital or ocular trauma;
7. Previous ocular surgery;
8. Acute or chronic, currently exacerbated respiratory diseases;
9. Decompensated cardiovascular diseases (e.g., a second- or third-degree atrioventricular block or cardiogenic shock);
10. Decompensated diabetes mellitus;
11. History of neurological disorders or mental illnesses.

### 2.2. Data Collection

**Figure 1:**
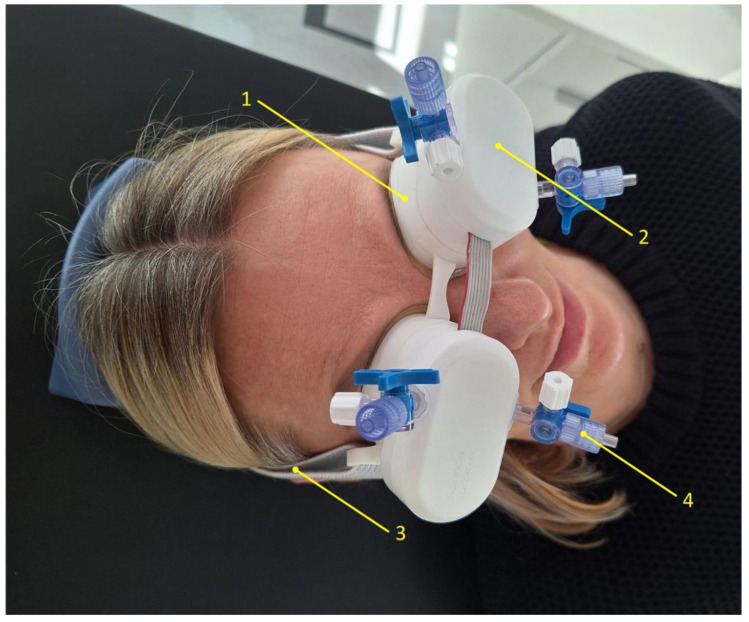
An image of an ‘Archimedes 02’ monitor placed on both closed eyelids of a control subject. 1—Chamber filled with a non-compressible liquid, 2—sensor assembly containing electronic components and a highly sensitive pressure transducer, 3—headband securing the device to the head, 4—valve for connecting a tube to fill the chamber with liquid and remove air.

Non-invasive monitoring of pressure pulse waves from closed eyelids was conducted using a recently developed technology called ‘Archimedes 02’, designed to monitor intracranial pressure waves [24]. The device is gently attached to both closed eyelids and secured with a band around the back of the head. The component in contact with the eyelids consists of a thin (50 µm) non-allergenic elastic film, which transmits pulsations from the closed eyelids to a non-compressible liquid. These pulsations are captured by a highly sensitive digital pressure sensor, which is in direct contact with the liquid. The baseline pressure of the liquid was set to 2.5 mmHg in both eyes of each subject before the monitoring session. Figure 1 shows the device placed on both closed eyelids of a control subject, prepared for intracranial pressure pulse wave monitoring. Pressure pulse wave monitoring was conducted for up to 5 min for each participant. The recorded signals were subsequently processed and analyzed using MATLAB (R2024a, MathWorks, Natick, MA, USA) to calculate the amplitude of the pressure pulse waves.

On the day of the examination, the study objectives, methods, and procedures were explained to all participants, who then provided written informed consent. All participants were in a supine position during the procedure, and all examinations were conducted during the daytime, between 8:00 a.m. and 7:00 p.m.

### 2.3. Statistical Analysis

Statistical analysis was performed using IBM SPSS Statistics software (version 30.0; IBM Corporation, Armonk, NY, USA). Two parameters—intraocular pressure and the amplitude of pressure pulse waves—were compared between the normal-tension glaucoma and control groups. The comparisons were conducted first by analyzing the right and left eyes separately, and then by including measurements from both eyes together. For the analysis of data from both eyes, a mixed ANOVA using the General Linear Model (GLM) procedure was applied to assess the effects of both between-group factors (control and NTG) and within-subject factors (left and right eyes). The Kolmogorov–Smirnov test was used to examine the data distribution normality. The analysis of the quantitative variables involved calculating the mean and standard deviation (SD), as well as the median and interquartile range (IQR). To compare the groups, Student’s *t*-test was used when the data were normally distributed, and the non-parametric Mann–Whitney U test was applied when the data did not follow a normal distribution. The significance level was set at *p* < 0.05.

## 3. Results

**Figure 2:**
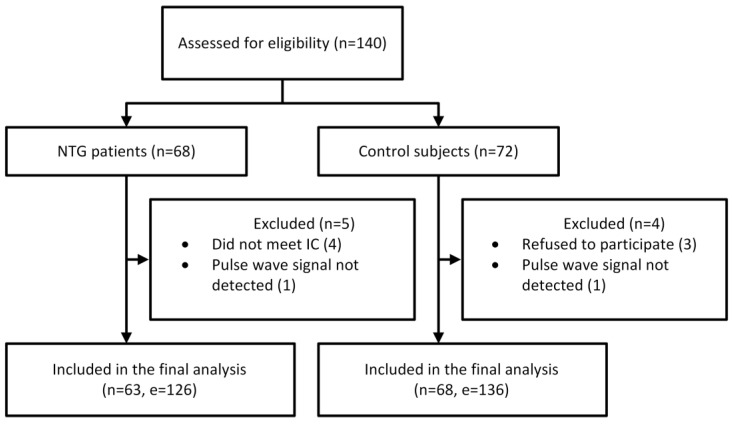
Flow chart of the study. Abbreviations: NTG, normal-tension glaucoma; IC, inclusion criteria; n, number of participants; e, number of eyes.

A total of 140 participants were enrolled in the study, comprising 68 NTG patients and 72 control subjects. A complete flow chart of the study is presented in Figure 2.

Following the exclusion, 63 NTG patients and 68 control subjects were included in the statistical analysis. The demographic characteristics of the participants are presented in Table 1, while the medical data are provided in Table 2.

**Figure 3:**
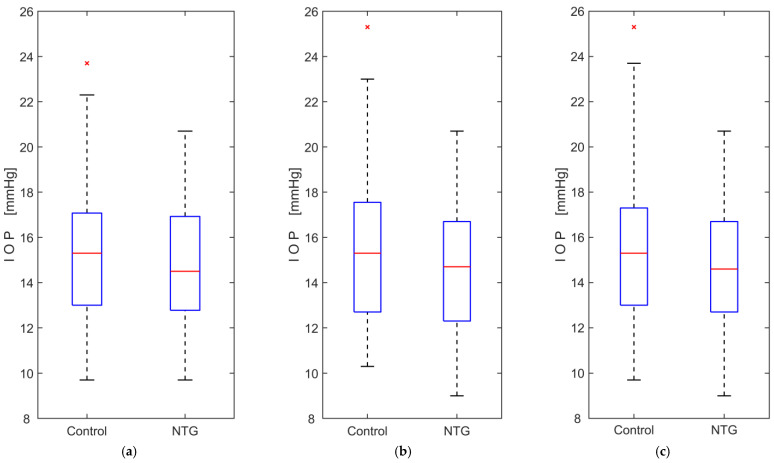
Boxplots of intraocular pressure (IOP) measurements comparing normal-tension glaucoma (NTG) patients and control subjects: (**a**) measurements from the left eye only; (**b**) measurements from the right eye only; (**c**) measurements from both eyes. The red lines represent the medians, while the red crosses (×) indicate statistical outliers.

The intraocular pressure data met the assumption of normality, according to the Kolmogorov–Smirnov test, for both groups in each case, with the right and left eyes analyzed separately and then together. The results of Student’s *t*-test showed no significant difference in IOP between the groups when comparing the right and left eyes separately. Analysis of IOP data showed no significant interaction between groups and eyes (F(1, 129) = 2.214, *p* = 0.139), no significant within-subject effect (left and right eyes; F(1, 129) = 2.311, *p* = 0.133), and no significant between-subjects effect (NTG and control groups; F(1, 129) = 2.242, *p* = 0.137). The results of the statistical analysis are presented in Table 3, and the boxplots of the IOP measurements are shown in Figure 3.

**Figure 4:**
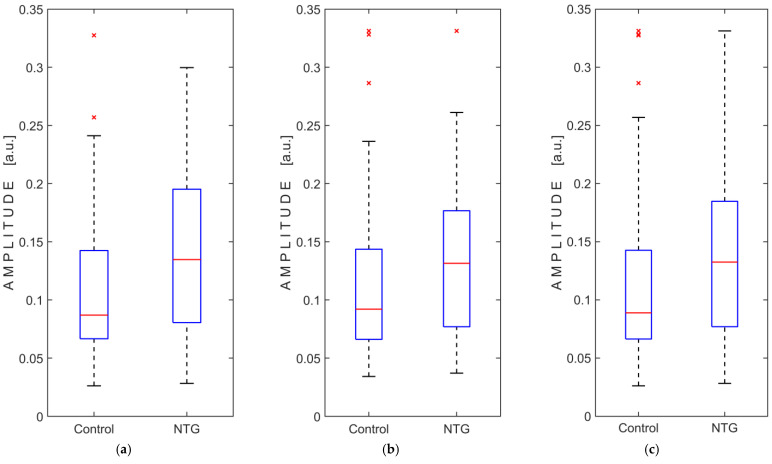
Boxplots of pressure pulse wave amplitude measurements comparing normal-tension glaucoma (NTG) patients and control subjects: (**a**) measurements from the left eye only; (**b**) measurements from the right eye only; (**c**) measurements from both eyes. Abbreviation: a.u., arbitrary units. The red lines represent the medians, while the red crosses (×) indicate statistical outliers.

The registered intracranial pressure pulse wave amplitude data did not meet the assumption of normality, as confirmed by the Kolmogorov–Smirnov test, for both groups in each case, with the right and left eyes were analyzed separately and then combined. A non-parametric Mann–Whitney U test was used to compare the groups and revealed statistically significant differences in amplitude between the NTG and control groups, both when the right and left eyes were compared separately and when both eyes were analyzed together. Analysis of amplitude data showed no significant interaction between groups and eyes (F(1, 129) = 1.196, *p* = 0.276), no significant within-subject effect (left and right eyes; F(1, 129) = 0.001, *p* = 0.978), but a significant main effect between subjects (NTG and control groups; F(1, 129) = 6.901, *p* = 0.01). The results of the statistical analysis are presented in Table 4, and the boxplots of the amplitude measurements are shown in Figure 4.

## 4. Discussion

Although the condition now termed normal-tension glaucoma was first described in the mid-19th century and its underlying mechanisms have been extensively explored in recent decades, they remain incompletely understood, and the disease continues to be significantly underdiagnosed [15,21,27]. A simple screening tool for evaluating normal-tension glaucoma would be valuable for ophthalmologists.

Researchers led by Singh et al. conducted a study measuring the pulsatile movement of the optic nerve head and the peripapillary retina using a novel Fourier-domain optical coherence tomography system [23]. They found that the mean fundus pulsation amplitude on the nasal side of the optic disc was significantly greater in glaucoma patients compared to normal subjects.

We recently developed non-invasive intracranial pressure wave monitoring technology [24]. This technology is based on the hypothesis that cerebrospinal fluid pulsations in the retrolaminar space along the optic nerve are transmitted to the outer ocular structures, where they can be detected through the closed eyelid as subtle pressure changes. In this paper, we investigated the amplitude of pressure pulse waves recorded from closed eyelids using the ‘Archimedes 02’ device in patients with normal-tension glaucoma and control subjects.

Subjects diagnosed with NTG by an ophthalmologist prior to our study were recruited for the study group, while the control group consisted of subjects with no history or diagnosis of glaucoma. We found that the mean intraocular pressure, measured in both eyes on the day of the examination, was 14.62 mmHg (SD = ±2.85 mmHg) for the NTG group and 15.39 mmHg (SD = ±3.16 mmHg) for the control group. The observed mean IOP for both groups fell within the normal range of 10–21 mmHg, which is considered typical for healthy subjects and NTG patients with glaucomatous damage [4,28].

This observational study showed a statistically significant difference (*p* = 0.01) in the amplitude of intracranial pressure pulse waves recorded from both closed eyelids, comparing the NTG group (median amplitude: 0.1326 a.u.) and the control group (median amplitude: 0.0889 a.u.). A statistically significant difference between groups was also observed when the recorded amplitude was analyzed separately, first for the left eye (*p* = 0.003) and then for the right eye (*p* = 0.016).

Several limitations of our study are worth mentioning. The gender ratio was skewed, with females comprising 75% of the control group and 79.4% of the NTG group. A larger sample size of male participants is needed to investigate potential differences in pressure pulse wave amplitude between genders. The greater proportion of female subjects enrolled in this study might be explained by findings from other studies indicating that normal-tension glaucoma is more common in women, or by the fact that females tend to live longer than males [14]. Age is epidemiologically considered to be a risk factor for NTG, and the likelihood of developing the disease increases with age [14,29]. The mean age of normal-tension glaucoma patients reported in many studies is in the 60s, which is consistent with the mean age of NTG patients (66 years) observed in this study [14,29]. However, at this age, individuals are often affected by many other health conditions. In this study, both the patients with normal-tension glaucoma and the control group had additional health conditions, such as high blood pressure, diabetes, heart disease, high cholesterol, and others. These conditions were treated with medications that affect the whole body (systemic medications). The influence of specific diseases or medications on the pressure pulse waves recorded using the technology in this study is not yet known.

While the results of this study demonstrate a significant difference in intracranial pressure pulse wave amplitude between NTG patients and controls, it is important to acknowledge that these findings do not yet establish the new measurement method as a replacement for existing clinical practices, such as slit-lamp examinations and tonometry. The ‘Archimedes 02’ technology shows promise as a potential supplementary tool, but further research and validation are necessary to confirm its clinical utility and diagnostic accuracy. Consequently, its role in routine ophthalmological practice remains exploratory at this stage.

In addition to its potential as a supplementary diagnostic tool, the ‘Archimedes 02’ technology may offer a foundation for individualized diagnostic support in the future. Although the current experimental results do not allow for its immediate application at the individual level, the ability to reflect personal physiological characteristics presents an intriguing avenue for further investigation. Additional studies involving diverse patient groups are essential to better understand individual differences in measurement results.

The findings of this observational study highlight the need for a prospective clinical investigation into the potential of ‘Archimedes 02’ as a screening tool for normal-tension glaucoma. Future research should focus on establishing a threshold for pulse wave amplitude to distinguish between healthy individuals and those with NTG. Subsequently, a randomized, double-blinded study involving both controls and NTG patients diagnosed by an ophthalmologist will be essential to assess the diagnostic accuracy, sensitivity, and specificity of ‘Archimedes 02’. Such studies will be crucial for determining whether this technology can evolve from a promising concept to a reliable tool of clinical practice.

## 5. Conclusions

In this study, we demonstrated that non-invasive technology designed to monitor intracranial pressure waves can detect pressure pulse waves through the closed eyelid in normal-tension glaucoma patients, and that these waves have a significantly greater amplitude in NTG patients compared to control subjects. The role of this technology in screening for normal-tension glaucoma needs to be further investigated.

## Figures and Tables

**Table 1:** Demographic data of the included participants in this comparative study.

Group	Age, Years				Gender			
	Mean	±SD	Min	Max	Male, n	Female, n	Male, %	Female, %
Control	58.91	±12.76	25	84	17	51	25.0	75.0
NTG	66.63	±9.86	43	85	13	50	20.6	79.4

Abbreviations: NTG, normal-tension glaucoma; SD, standard deviation; n, number of participants.

**Table 2:** Medical data of the participants included in this comparative study.

Group	Control	NTG
NTG medication, n	No medication, 68	No medication, 6 Pg analogues, 47 CAIs, 19 β blockers, 30 α2 agonists, 2
Comorbidity, n	No comorbidities, 38 Arterial hypertension, 27 Diabetes mellitus, 4 Hypothyroidism, 3 Depression, 2 Gout (podagra), 3 Rheumatoid arthritis, 1 Psoriasis, 1 Asthma, 1 Ischemic heart disease, 3 Gastritis, 1	No comorbidities, 23 Arterial hypertension, 33 Heart failure, 4 Hypercholesterolemia, 4 Parkinson’s disease, 1 Autoimmune thyroiditis, 1 Diabetes mellitus, 3 Depression, 1 Gout (podagra), 2 Osteoporosis, 1 Angina pectoris, 2 Chronic atrial fibrillation, 3 BPH, 1 Hyperlipidemia, 1
Systemic medication, n	No systemic medication, 41 CCB, 9 ACE inhibitor, 11 Thyroid hormone, 2 Statin, 10 β-blockers, 13 SSRI, 2 XDH inhibitor, 2 Thiazide-like diuretic, 2 Biguanide, 2 ARB, 3 T4 hormone, 2 Other, 11	No systemic medication, 22 ARB, 6 Thiazide diuretic, 2 Statin, 11 β-blockers, 22 ACE inhibitor, 13 BDZ, 3 NSAID, 6 SIRAs, 2 α2-agonist, 2 Thiazide-like diuretic, 2 XDH inhibitor, 2 Biguanide, 2 CCB, 6 Xa inhibitor, 3 Other, 14

Abbreviations: NTG, normal-tension glaucoma; CS, control subjects; n, number of participants; Pg analogues, prostaglandin analogues; CAIs, carbonic anhydrase inhibitors; BPH, benign prostatic hyperplasia; ARB, angiotensin receptor blocker; ACE inhibitor, angiotensin-converting enzyme inhibitor; BDZ, benzodiazepine; NSAID, non-steroidal anti-inflammatory drug; SIRAs, selective imidazoline receptor agonists; XDH inhibitor, xanthine dehydrogenase inhibitor; CCB, calcium channel blocker; Xa inhibitor, factor Xa inhibitor; SSRI, selective serotonin reuptake inhibitor.

**Table 3:** Results of statistical tests for IOP measurements.

Analyzed Eyes	Left		Right		Both	
Group	Control	NTG	Control	NTG	Control	NTG
Mean (±SD)	15.39 (±3.20)	14.80 (±2.78)	15.39 (±3.15)	14.43 (±2.93)	15.39 (±3.16)	14.62 (±2.85)
Median (IQR)	15.15 (13.00–17.22)	14.50 (12.70–17.00)	15.30 (12.70–17.65)	14.70 (12.30–16.70)	15.30 (12.78–17.30)	14.60 (12.70–16.70)
K-S test *p*-value	0.200	0.200	0.200	0.200	0.052	0.200
Significance between groups	t = 1.112, df = 129, *p* = 0.268		t = 1.795, df = 129, *p* = 0.075		F(1, 129) = 2.242, *p* = 0.137	

Abbreviations: IOP, intraocular pressure; NTG, normal-tension glaucoma; SD, standard deviation; IQR, interquartile range; K-S test, Kolmogorov–Smirnov test; t, Student’s *t*-test statistic; df, degrees of freedom; F, F-statistic from the mixed ANOVA test, used to assess between-subjects effects.

**Table 4:** Results of statistical tests for pulse wave amplitude measurements.

Analyzed Eyes	Left		Right		Both	
Group	Control	NTG	Control	NTG	Control	NTG
Mean (±SD)	0.1106 (±0.0746)	0.1539 (±0.1068)	0.1176 (±0.0771)	0.1466 (±0.0875)	0.1141 (±0.0757)	0.1503 (±0.0973)
Median (IQR)	0.0869 (0.0664–0.1433)	0.1347 (0.0798–0.1967)	0.0921 (0.0659–0.1447)	0.1315 (0.0769–0.1775)	0.0889 (0.0663–0.1434)	0.1326 (0.0769–0.0185)
K-S test *p*-value	<0.001	0.007	<0.001	0.006	<0.001	<0.001
Significance between groups	U = 1501.0, Z = −2.953, *p* = 0.003		U = 1620.4, Z = −2.402, *p* = 0.016		F(1, 129) = 6.901, *p* = 0.01	

Abbreviations: NTG, normal-tension glaucoma; SD, standard deviation; IQR, interquartile range; K-S test, Kolmogorov–Smirnov test; U, Mann–Whitney test U statistic; Z, Mann–Whitney statistics Z score; F, F-statistic from the mixed ANOVA test, used to assess between-subjects effects.

## Data Availability

Due to privacy concerns and ethical considerations, access to the clinical data used in this study is restricted. The data are available upon reasonable request, subject to approval by the Regional Kaunas Biomedical Research Ethics Committee (kaunorbtek@lsmuni.lt).
